# Silencing of the CrkL gene reverses the doxorubicin resistance of K562/ADR cells through regulating PI3K/Akt/MRP1 signaling

**DOI:** 10.1002/jcla.23817

**Published:** 2021-06-11

**Authors:** Jiang Yu, Wen‐XU Chen, Wen‐Jing Xie, Rong‐Wei Chen, Dan‐Qi Lin, Wei‐Wei You, Wei‐Lin Ye, Hong‐Qin Zhang, Dong‐Hong Lin, Jian‐Ping Xu

**Affiliations:** ^1^ Department of Clinical Laboratory Fuzhou Second Hospital Affiliated to Xiamen University Fuzhou Fujian China; ^2^ Department of Pharmacy clinical Pharmacy Fuzhou Second Hospital Affiliated to Xiamen University Fuzhou Fujian China; ^3^ Department of Clinical Laboratory Medicine Fujian Medical University Fuzhou China

**Keywords:** CrkL, doxorubicin resistance, MRP1, myelogenous leukemia

## Abstract

**Background:**

Doxorubicin is a first‐line chemotherapy agent on human myelogenous leukemia clinical treatment, but the development of chemoresistance has largely limited curative effect. In this study, we aimed to evaluate the biological function and molecular mechanisms of CrkL to Doxorubicin resistance.

**Methods:**

Quantitative reverse transcription‐PCR (qRT‐PCR) assay was performed to examine the expression of CrkL in K562 and K562/ADR cells. The expression of CrkL was silenced through RNA interference technology. MTT assay and flow cytometry were performed to detect the proliferation inhibition and apoptosis rate after CrkL siRNA transfection. The protein expression changes of PI3K/AKT/MRP1 pathway induced by CrkL siRNA were observed by Western Blot assay. Xenograft tumor model was carried out to observe tumor growth in vivo.

**Results:**

We observed that silencing of CrkL could effectively increase apoptosis rate induced by doxorubicin and dramatically reversed doxorubicin resistance in K562/ADR cells. Further studies revealed knockdown CrkL expression suppressed PI3K/Akt/MRP1 signaling, which indicated CrkL siRNA reversed doxorubicin effect through regulating PI3K/Akt/MRP1 pathway. In addition, overexpression of MRP1 could evidently reduce apoptosis rate and reversed the inhibitory effects of doxorubicin resistance caused by CrkL siRNA on K562/ADR cells. Finally, in vivo experiments revealed that CrkL silencing acted a tumor‐suppressing role in myelogenous leukemia via regulating PI3K/Akt/MRP1 signaling.

**Conclusion:**

Together, we indicated that CrkL is up‐regulated in myelogenous leukemia cells and silencing of CrkL could reverse Doxorubicin resistance effectively. These results show a potential novel strategy for intervention chemoresistance in myelogenous leukemia during chemotherapy.

## Graphical abstract

Doxorubicin is a first‐line chemotherapy agent on human myelogenous leukemia clinical treatment, but the development of chemoresistance has largely limited curative effect. In this study, we aimed to evaluate the biological function and molecular mechanisms of CrkL to Doxorubicin resistance. We observed that silencing of CrkL could effectively increase apoptosis rate induced by doxorubicin and dramatically reversed doxorubicin resistance in K562/ADR cells. Further studies revealed knockdown CrkL expression suppressed PI3K/Akt/MRP1 signaling, which indicated CrkL siRNA reversed doxorubicin effect through regulating PI3K/Akt/MRP1 signaling. In addition, overexpression of MRP1 could evidently reduce apoptosis rate and reversed the inhibitory effects of doxorubicin resistance caused by CrkL siRNA on K562/ADR cells. In vivo experiments revealed that CrkL silencing suppressed tumor growth of K562/ADR through regulating PI3K/AKT/MRP1 signaling.Together, we indicated that CrkL is up‐regulated in myelogenous leukemia cells and silencing of CrkL could reversed Doxorubicin resistance effectively. These results show a potential novel strategy for intervention chemoresistance in myelogenous leukemia during chemotherapy.

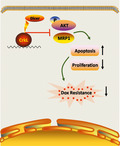

## 1 INTRODUCTION

Leukemia is one of most frequent types of human cancers worldwide. It can be divided into acute and chronic leukemia according to the degree of differentiation and the length of the natural course. On the basis of the types of blood cells affected, it can be divided into myeloid and lymphoblastic leukemia.^1^ Chemotherapy is the crucial approach to treat leukemia clinically and Doxorubicin (Dox) is the most commonly used chemo‐therapeutic agent.^2^ Dox is an anthracycline‐based anti‐tumor drug and kills leukemia cells by inhibiting DNA transcription and RNA synthesis, and also destroys the DNA and protein structures of leukemia cells.^3^ But the occurrence of chemoresistance and side effects seriously impacts the treatment efficacy and shortens patients’ survival time. Therefore, a great deal of attention has been focused on identifying the genetic and molecular mechanisms of Dox resistance. Until now, series of chemoresistance research revealed the ATP‐binding cassette (ABC) transporter superfamily contributed to multi‐drug resistance(MDR),^4^ which contained the P‐glycoprotein (P‐gp),MRP1(multi‐drug‐associated resistance protein 1) and ABCC1 (ATP‐Binding Cassette Subfamily C Member 1) transporter. MRP1 utilizes the energy from ATP‐binding or hydrolysis and actively effluxes intracellular drug out of cells leading to MDR.^5^, ^6^

CrkL is a member of the Crk adapter protein family, which is involved in two alternatively spliced isoforms of CRK(Crk I and Crk II), human homolog of avian sarcoma retroviral v‐crk oncogene and Crk‐like protein (CrkL).^7^ Recent studies have demonstrated that CrkL acts a pivotal part in cell proliferation and associated with the migration and invasion in range of cancers such as bladder,^8^ breast cancers,^9^ lung,^10^ stomach,^11^ and liver.^12^ The high expression level of CrkL has been related to advanced‐stage cancers with high‐grade aggressiveness and poor prognosis. Cheung et al.^10^ found that overexpression of CRKL triggered gefitinib resistance in epidermal growth factor receptor (EGFR)‐mutant cells via actuating AKT signaling and extracellular signal‐regulated kinase in non‐small cell lung cancer. The high expression of CrkL intervened CCL20/CCR6‐induced EMT through Akt signaling to the occurrence and development of gastric cancer.^13^ The studies elucidated that CrkL contributed to the amplification and initiation of oncogenic signals to promote tumorigenesis. However, the function and molecular mechanism underlying CrkL expression in non‐solid malignancies are largely undiscovered.

In the present study, the main objective was to determine the biological function of CrkL on Dox resistance of myelogenous leukemia and to find the molecular mechanism by which it occurs. We found that silencing CrkL expression reversed the Dox resistance of K562/ADR cells by regulating PI3K/Akt/MRP1 signaling in vitro and in vivo. MRP1 overexpression reversed the inhibition of CrkL siRNA. Our findings bring forth new insights into the role of CrkL on Dox resistance to myelogenous leukemia, and further studies are needed to confirm CrkL as a potential therapeutic target for myelogenous leukemia patients with chemoresistance.

## 2 MATERIALS AND METHODS

### 2.1 Reagents and cell culture

Doxorubicin (Dox) purchased from Sigma‐Aldrich was dissolved in sterile double distilled water at a concentration of 3 g/L before experiment. Anti‐CrkL, anti‐p‐AKT, anti‐AKT, anti‐MRP1, and anti‐β‐actin monoclonal antibodies were purchased from Affinity Biosciences and Abcam Inc. MTT and Annexin Ⅴ‐FITC/PI Kit were obtained from 4A biotech. The human myelogenous leukemia K562 cells doxorubicin‐resistant counterpart K562/ADR cells were obtained from the Tongpai Biotechnology CO., LTD. K562 cells were cultured in RPMI‐1640 medium containing 10% fetal bovine serum and incubated at 37˚C in a 5% CO_2_ atmosphere. The culture medium of K562/ADR cells required an additional supplement of 1 mg/L Dox. K562/ADR cells need to be cultured in Dox‐free medium for 2 days before the experiments.

### 2.2 Cell transfection

The siRNA oligonucleotide targeting the human CrkL gene (sense: 5‐GUGAGAUCCUAGUGAUAAUUU‐3,anti‐sense:5‐UUCACUCUAGGAUCACUAUUA‐3) was designed and synthesized by Shanghai Genepharma Co. Ltd. as previous reports. Meanwhile, a nonspecific control siRNA was transfected into cells as a negative control. The MRP1 overexpression plasmid vector was constructed based on pcDNA3.1 vector (Invitrogen). MRP1 overexpression was induced via pcDNA3.1‐MRP1 transient transfection using Lipofectamine 2000 (Invitrogen). The empty pCDNA3.1 vector was tested as a control. Before transfection with siRNA (100 nM) with Lipofectamine, cells should be cultured in antibiotics‐free medium for 24 h. After the cells adhere and fusion rate up to 80%, the culture medium was replaced with Serum‐free RPMI‐1640 medium, and with 5 μg of Lipofectamine 2000 and 5 μl of CrkL siRNA or NC were added. For plasmid transfection, 5 μg of Lipofectamine 2000 and 2 μg of pcDNA3.1‐MRP1 or pCDNA3.1 vector were added. It needed to recover to a serum‐containing medium after action for 6 h.

### 2.3 Drug sensitivity assay

The cytotoxicity of Dox was examined through MTT assay in K562 and K562/ADR cells. Cells were seeded into 96‐well plates (4 × 10^5^ cells/well) and cultured for 24 h at 37°C and 5% CO_2_. The cells were then exposed to graded concentrations of DOX in K562 cells (0.1 μg/mL, 0.2 μg/mL, 0.4 μg/mL, 0.8 μg/mL, and 1.6 μg/mL) and K562/ADR cells (2.5 μg/mL, 5.0 μg/mL, 10.0 μg/mL, 20.0 μg/mL, and 40.0 μg/mL) for 24 h at 37°C and 5% CO_2_ (three replicates for each concentration). 10 μL of MTT solution was added to every well, and then plates were cultured for another 4 h. The absorbance was measured three times at 450 nm wavelength by a microplate reader, and the growth inhibition curves were established through average absorbance values. Cell inhibitory ratio (%) = (A450_control_−A450_sample_)/(A450_control_−A450_blank_)×100%. The half inhibitory concentration (IC_50_) of Dox was calculated as previously described.^14^ Reversal Fold(RF) = (K562 /ADR IC_50_/(K562/ADR CrkL siRNA IC_50_).

### 2.4 Apoptosis assay

Cell apoptosis assay was carried out using AnnexinⅤ‐FITC/PI Kit according to the manufacturer's instructions. Briefly, cells were treated for 48 h and harvested. After washing three time with pre‐cooled PBS, the cells were resuspended in 0.5 mL binding buffer. And then 10 µL propidium iodide and 10 µL Annexin‐V‐FITC were further added into the system for 15 min incubation. Finally, the cells were washed to remove excess stains and detected via the flow cytometer (BD Biosciences).

### 2.5 Quantitative real‐time PCR assay

Total RNA was extracted using Trizol reagent (Invitrogen) and then reversely transcribed to cDNA using a PrimeScript RT Reagent kit (Takara). 25 μL reaction mixture was consisted with 12.5 μL of SYBR FAST master mix, 1 μL aliquot of aptamer‐containing solution, 0.5 μL of reverse primer and nuclease‐free water. qRT‐PCR was carried out to initial denaturation for 2 min at 95°C followed by cycling at 95°C for 30 s, and then annealing/extension for 20 s at 72°C. This process was repeated on PCR machine for 40 times. Data were analyzed using the comparative Ct method (2−ΔΔCt). The primers used for real‐time PCR were as listed in Table 1.

**TABLE 1:** The primers of real‐time PCR

Gene	Forward	Reverse
CrkL	5'‐CGCTCCGCCTGGTATATGG‐3'	5'‐GGACACCGACAGCACATAGTC‐3'
MRP1	5'‐GGACTTCGTTCTCAGGCACA‐3'	5'‐TGCAGTCCTCGAACTGTGTC‐3'
β‐actin	5'‐GACAGGATGCAGAAGGAGATTACT‐3'	5'‐TGATCCACATCTGCTGGAAGGT‐3'

### 2.6 Western blot analysis

Cells were washed in cold PBS buffer for three times and lysed in ice‐cold RIPA buffer for 30 min. Cell debris was removed through centrifugation at 12,000 rpm for 10 min at 4°C. The protein concentration was verified via the BCA protein quantitation kit. Protein samples were electrophoresed on SDS‐PAGE and then transferred to PVDF membranes. 5% skim milk was used to block the nonspecific binding at room temperature for 2 h. The membranes were immunoblotted with primary monoclonal antibodies against CrkL (1:1000), p‐AKT (1:1000), AKT (1:500), MRP1(1:1000), and β‐actin (1:1500) overnight at 4°C and followed by washing the membranes three times for 5 min with TBST (TBS containing 0.1% Tween‐20) buffer. Then the membranes were incubated at room temperature for 2 h with horseradish peroxidase (HRP)‐conjugated secondary antibody (1:1000) and then cultured with 10 mL ECL (Amersham) for 2 min. The protein bands were quantified and analyzed through ImageJ software.

### 2.7 Xenograft tumor model

K562/ADR cells were harvested and resuspended in RPMI‐1640 medium. 15 nude mice were injected with a total number of 3 × 10^6^/100 μL cells subcutaneously at the posterior flank. Tumor size was monitored by measuring the length (L) and width (W) with calipers every 7 days. All the mice were euthanized through intraperitoneal injection of excessive pentobarbital sodium (100 mg/kg) after 35 days. Subsequently, the tumors were excised out from the sacrificed mice and weighed. Animal experiments were performed totally according to the Guide for the Care and Use of Laboratory Animals.

### 2.8 Immunohistochemistry

The tumor tissues were fixed in 4% paraformaldehyde for 24 h, dehydrated in a graded alcohol series and embedded in paraffin followed by cutting into 5 μm sections. The sections were deparaffinized, rehydrated with a graded alcohol series, and then incubated in 96℃ with 0.01 mol/L sodium citrate buffer for the antigen retrieval. After incubation in 5% H_2_O_2_ for 2 h, the sections were incubated with Ki67 primary antibodies overnight at 4℃. Immunostaining was performed using streptavidin‐peroxidase and diaminobenzidine (DAB) according to the manufacturer's instructions (Beyotime). Finally, the sections were observed under a fluorescence microscope (Leica) and pictured.

### 2.9 Statistical analysis

Statistical analyses were carried out using the SPSS 19.0. Continuous variables were expressed as the mean ± standard deviation from at least three separate experiments. Differences were analyzed by Student's t test between two groups or one‐way analysis of variance (ANOVA) among multiple groups. *p* values <0.05 were considered statistically significant.

## 3 RESULTS

### 3.1 CrkL was up‐regulated in human myelogenous leukemia cells

**FIGURE 1:**
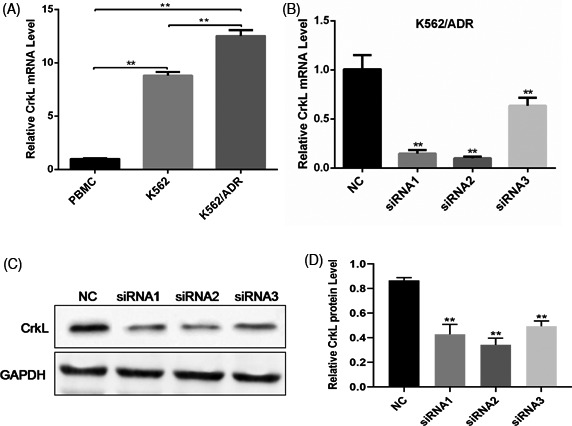
CrkL is up‐regulated in myelogenous leukemia cells. (A) Expression of CrkL gene in K562 and K562/ADR was detected by qRT‐PCR. CrkL was significantly up‐regulated in myelogenous leukemia cells compared with PBMC cells. The inhibition efficiency of specific CrkL small interference RNA (siRNA) in K562/ADR cells was measured by qRT‐PCR (B) and Western Blot (C‐D). The data were expressed as the mean ± SD of three independent experiments. *p < .05, **p < 0.01

To investigate the role of CrkL in human myelogenous leukemia, we explored the expression of CrkL in myelogenous leukemia cells (K562 and K562/ADR) and PBMC cells by qRT‐PCR analysis. The results showed that CrkL gene was significantly up‐regulated in myelogenous leukemia cells compared with PBMC cell. Notably, compared with K562 cells, CrkL expression was obviously higher in K562/ADR cells (Figure 1A). The interference efficiency of CrkL was determined through qRT‐PCR and Western Blot. The results revealed that the CrkL expression was obviously weakened in CrkL siRNA1 and siRNA2 groups compared with the negative control (Figure 1B‐D). The CrkL siRNA2, which showed the greatest interference effect, was selected for follow‐up experiments.

### 3.2 CrkL silencing reduced the doxorubicin resistance and increased apoptosis in K562/ADR cells

**FIGURE 2:**
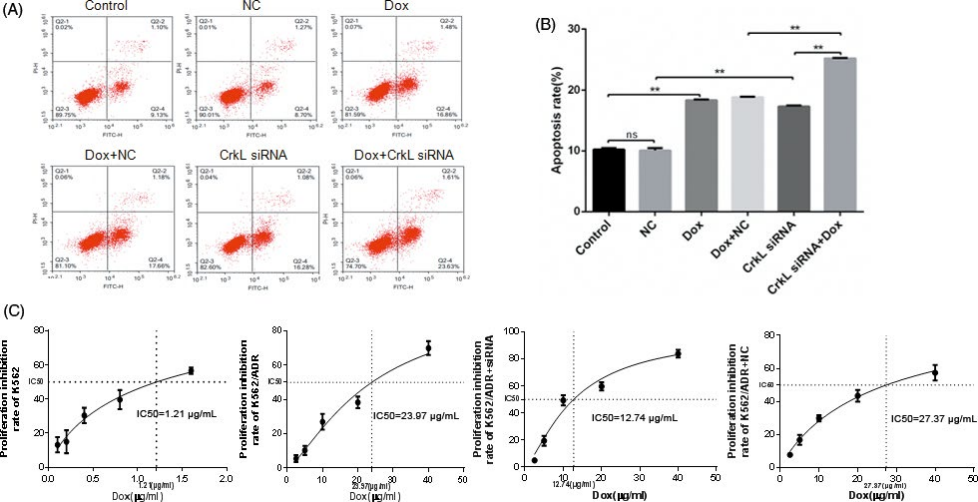
CrkL knockdown reduces Dox resistance and increases apoptosis in K562/ADR cells. (A) Cells were treated with the indicated concentrations of Dox for 48 h in the absence or presence of CrkL siRNA and then subjected to Annexin‐V assay. (B) CrkL siRNA increased the numbers of apoptotic K562/ADR cells. (C) Cells were treated with different concentrations of Dox in the absence or presence of CrkL siRNA. Cell viability was measured 48 h following treatment by MTT assay. CrkL siRNA effectively reduced proliferation inhibition of K562/ADR cells. The data were expressed as the mean ± SD of three independent experiments. *p < 0.05, **p < 0.01, ns means not significant, *p > *0.05

We examined whether CrkL silencing sensitizes K562/ADR cells to Dox. The MTT assay determined the growth inhibition rate after cultured with a range of concentrations of Dox for 48 h. As shown in (Figure 2C), compared with K562 cells, K562/ADR cells showed poor response to Dox. Remarkably, the K562/ADR/CrkL siRNA cells showed significantly greater proliferation suppression than the K562/ADR/NC and the negative control group. The absorbances were used to calculate IC50 values of Dox to reveal whether silencing of CrkL promoted Dox sensitivity in the K562/ADR cells. The IC50 values of Dox in the K562/ADR cells were 1.21, 23.97, 12.74, and 27.37 µg/mL for K562, K562/ADR, K562/ADR/CrkL siRNA, and K562/ADR/NC groups, respectively. The reversal fold‐change of K562/ADR/CrkL siRNA was 1.88, respectively (Table 2). Subsequently, flow cytometry analysis was performed to further determine the effect of CrkL on Dox‐induced apoptosis. The results revealed that CrkL silencing dramatically enhanced Dox‐induced apoptosis in K562/ADR cells (Figure 2A‐B). Collectively, silencing of CrkL improved Dox sensitivity in K562/ADR cell.

**TABLE 2:** Effect of CrkL silencing on the sensitivity of K562/ADR cells toward Dox by CCK‐8 assay

Treatment	IC50 ± SD^a^ (μg/mL)	RF^b^
Dox alone	23.97 ± 3.22	
NC	27.37 ± 2.06	
CrkL siRNA	12.74 ± 1.59^c^	1.88

^a^ IC50 values are represented as means ± SD of three independent experiments performed in triplicate.

^b^ RF, Resistance fold. RF = (K562 /ADR IC50)/(K562/ADR CrkL siRNA IC50).

c *p* < 0.05 versus the NC group.

### 3.3 Silencing of CrkL inhibited PI3K/Akt/MRP1 signaling in K562/ADR cells

**FIGURE 3:**
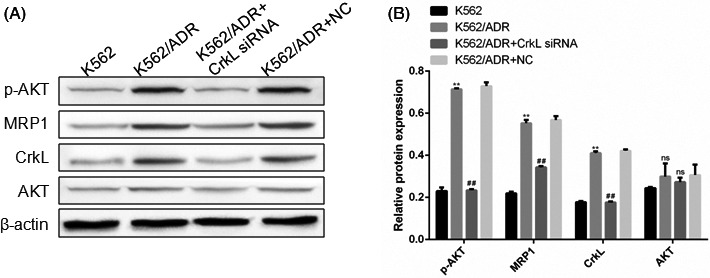
SILENCING OF CRKL INHIBITED PI3K/AKT/MRP1 PATHWAY IN K562/ADR CELLS. (A) CELLS Were left Dox‐untreated for 48 h for Western blot assay, measuring PI3K/AKT/MRP1 pathway‐related protein expression. (B) Bar diagram showed the relative expressions of proteins normalized to β‐actin. CrkL knockdown in K562/ADR cells could suppress p‐AKT, MRP1 protein levels while AKT expression remained unchanged compared with the control and NC groups. The data were represented as mean ± SD of three independent experiments. *p < 0.05, **p < 0.01, ns means not significant, *p *> 0.05

The PI3K/AKT/MRP1 signaling acts a pivotal part in cellular metabolism, cell proliferation and considered to be one of frequently mutated pathway in cancers. To reveal how CrkL enhances the Dox susceptibility to K562/ADR cells, we measured the protein expression of PI3K/AKT/MRP1 pathway after CrkL siRNA or NC transfection. The results showed that CrkL silencing in K562/ADR cells could suppress p‐AKT, MRP1 protein levels while AKT expression remained unchanged compared with the control and NC groups (Figure 3A‐B). The results indicate that CrkL might enhance the Dox susceptibility through down‐regulating PI3K/AKT/MRP1 signaling.

### 3.4 MRP1 overexpression reversed the inhibition of CrkL siRNA on Dox resistance to K562/ADR cells

**FIGURE 4:**
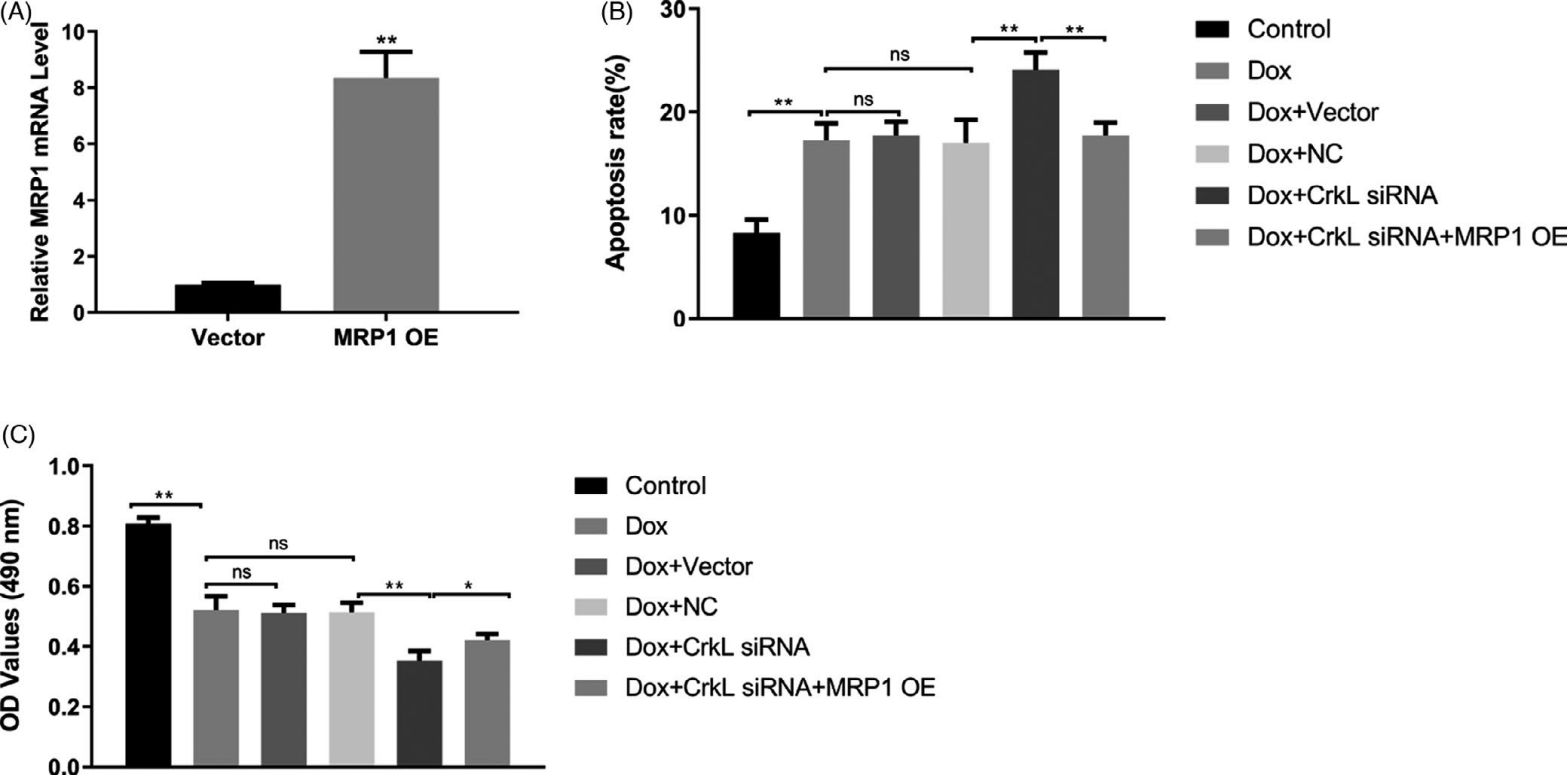
MRP1 overexpression reversed CrkL siRNA‐mediated inhibition to Dox resistance to K562/ADR cells. (A) qRT‐PCR analysis was carried out to detect the transfection efficiency following the overexpression of MRP1. (B) K562/ADR cells were transfected with either control or MRP1 overexpression plasmids for 48 h after CrkL siRNA treated. Then cells were subjected to flow cytometry assay. MRP1 overexpression decreased the apoptosis rate compared with CrkL siRNA alone transfection. (C) The CCK‐8 assay measured the effect of overexpression of MRP1 on proliferation of K562/ADR cells. MRP1 overexpression significantly reversed CrkL siRNA‐inhibited K562/ADR cells proliferation. The data were represented as mean ± SD of three independent experiments. *p < 0.05, **p < 0.01, ns means not significant, *p *> 0.05

MRP1 has been reported to play an essential role in chemoresistance. In this study, to further determine that CrkL affects Dox resistance to K562/ADR cells via PI3K/AKT/MRP1 pathway, MRP1 overexpression was transfected into cells after CrkL siRNA treated. Firstly, we constructed the MRP1 overexpression plasmids. The overexpression efficiency was determined by RT‐PCR analysis after transfected it in K562/ADR cells (Figure 4A). The results revealed that MRP1 upregulation could effectively reverse the proliferation inhibition of CrkL siRNA in K562/ADR cells (Figure 4C). In addition, flow cytometry analysis showed that MRP1 overexpression remarkably decreased the apoptosis rate compared with CrkL siRNA alone transfection (Figure 4B). These results further proved that CrkL reduced Dox resistance to K562/ADR cells through PI3K/AKT/MRP1 pathway.

### 3.5 CrkL silencing suppressed the tumor growth of K562/ADR cells in vivo

**FIGURE 5:**
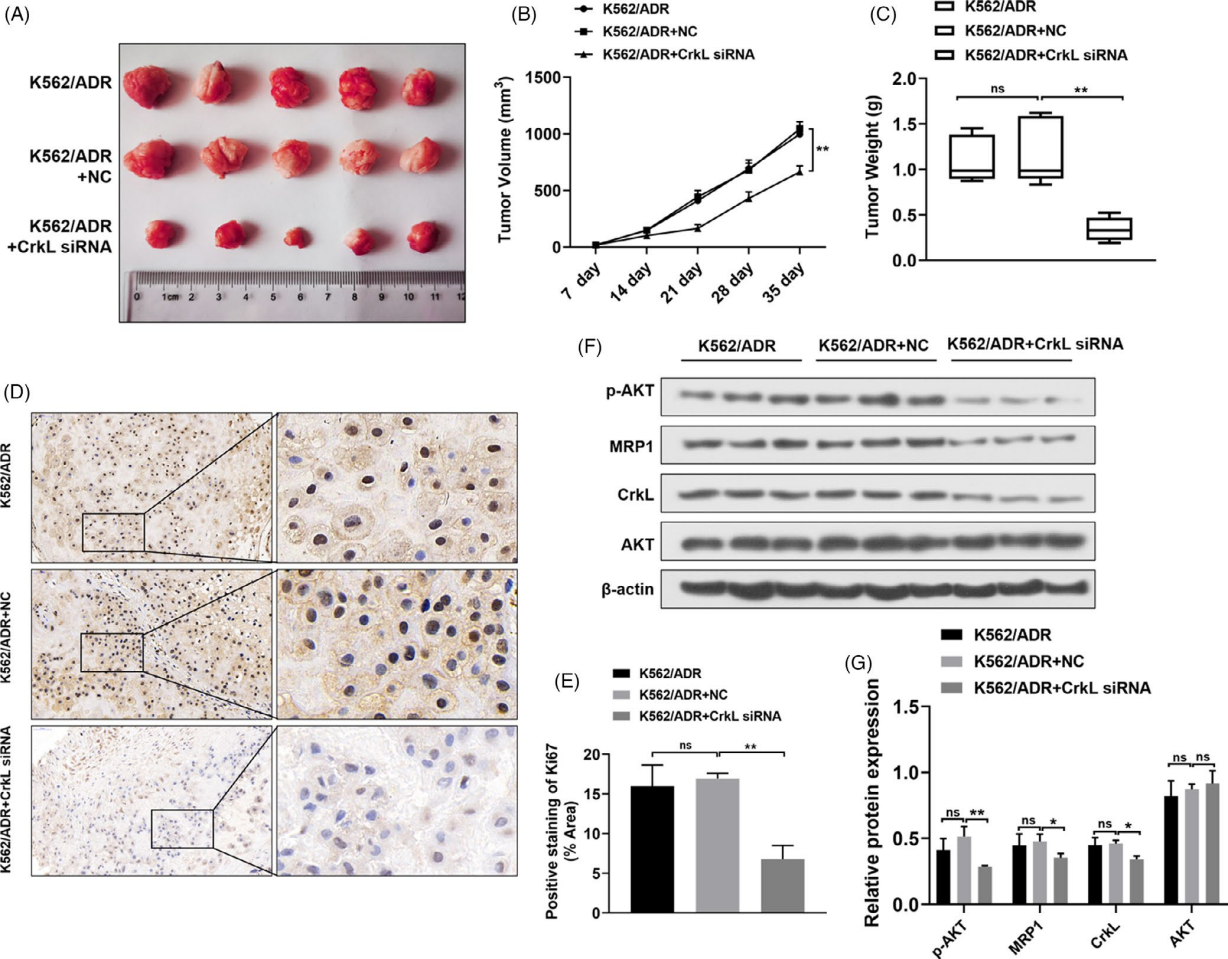
Effects of CrkL silencing on nude mice burdened with K562/ADR xenografts. A xenograft tumor model was established via subcutaneously injecting K562/ADR cells. (A) Tumor image was shown in each group. (B‐C). The growth curves and weights of the tumors in each group were measured. Silencing CrkL notably inhibited the tumor growth compared with NC group. (D‐E) The images of Ki67‐IHC were shown in each group and the Ki67 positive staining area was measured. The Ki67 positive staining area of CrkL siRNA treatment was lower compared with the NC group. (F‐G) Western blot assay was performed to determine PI3K/AKT/MRP1 pathway‐related proteins expression. CrkL silencing in vivo significantly suppressed p‐AKT, CrkL, MRP1 protein levels while AKT expression remained unchanged compared with the control and NC groups. The data were represented as mean ± SD of three independent experiments. *p < 0.05, **p < 0.01, ns means not significant, *p *> 0.05

Next, we carried out in vivo study to further investigate the biological effects of CrkL silencing in the progression of myelogenous leukemia. Stable K562/ADR cell line was established with transfection of CrkL knock down vectors and its control vector respectively. As the results showed, the tumor growth was inhibited by CrkL knockdown (Figure 5A‐B). And the weight of the tumors was reduced by CrkL siRNA treatment (Figure 5C). Ki67 is a nuclear protein associated with tumor cell proliferation and used widely as a marker to determine the tumor growth in routine pathological investigation.^15^ The IHC staining was applied to determine the Ki67 expression in tumor tissues and the results showed CrkL silencing could notably inhibit Ki67 expression compared with the vector group (Figure 5D‐E). Furthermore, Western Blot assay revealed that the expression level of p‐AKT, CrkL and MRP1 protein levels inhibited while AKT expression remained unchanged compared with the NC groups after CrkL siRNA treatment (Figure 5F‐G). Thus, we concluded that CrkL silencing inhibited the promotion of myelogenous leukemia in vitro and in vivo.

## 4 DISCUSSION

Human myelogenous leukemia is one of most common malignancies worldwide with characteristics of rapid progression, poor prognosis and high mortality. Chemotherapy is the primary clinical treatment. But the occurrence of drug resistance is the main obstacle during the long‐time treatment, which leads to less than expected curative effect.^16^ Therefore, it has grown attention on exploring the molecular mechanisms of Dox resistance and pursuing a novel target‐oriented therapy to conquer it.

There are mainly four molecular mechanisms of drug resistance to cancer treatment^17^, ^18^: (a) glutathione that could efflux drugs out of tumor cells; (b) p53 mutation suppresses apoptosis; (c) multiple drug resistance proteins, consist of MRP1, LRP, P‐gp, generally over‐expressed in drug resistance cells and these proteins restrain drug from entering the cell interior; and (d) tumor microenvironment alters. Upregulation of MRP1 is known to promote multi‐drug resistance to cancers.^19^ However, the mechanisms of MRP1 overexpression in myelogenous leukemia cells are still not fully clear. Previous studies have shown that the PI3K/AKT pathway was closely related to the development of cancers and acted a critical role in the occurrence of chemoresistance.^20^ In addition, it has been proved that the inhibitor of the PI3K/AKT pathway could decrease the expression of MRP1 and reverse the chemoresistance in colon carcinoma cells.^21^

CrkL is an adaptor protein involved of a single N‐terminal Src homology 2 (SH2) domain followed by two SH3 domains.^22^ The SH2 domain binds to a polyproline (PPII) peptides of pTyr‐x‐x‐Pro motif, while the SH3N domain intervenes the targets through a proline‐rich Pro‐x‐x‐Pro‐x‐(Lys, Arg) motif.^23^, ^24^ The high expression of CrkL improves adhesion‐independent proliferation in fibroblasts^25^ and promotes the morphological transformation of human airway epithelial cells by triggering SOS1/C3G/RAP1 signaling.^10^ Cheung et al.^10^ presented adequate genomic data certifying the amplification of CRKL gene at 22q11.21 in NSCLC and high expression of CRKL induces lung cancer cells with EGFR mutations resistant to EGFR inhibitors through regulating AKT and MAPK pathway. Zhang et al.^26^ reported that CRKL is one of the categories of PI3Kb‐interacting proteins. Knockdown CRKL expression in PTEN‐deficient cancer cells resulted in an enhances in p110b‐dependent PI3K signaling and cancer cell growth. In our study, K562/ADR cells were used to investigate the mechanisms of drug resistance to human myelogenous leukemia. We found CrkL was up‐regulated in human myelogenous leukemia cells and CrkL silencing effectively reduced the Dox resistance and increased apoptosis in K562/ADR cells. Further research revealed that the mechanism by which CrkL inhibited Dox resistance was through regulating PI3K/AKT/MRP1 pathway. MRP1 overexpression reversed the inhibition of CrkL siRNA on Dox resistance to K562/ADR cells.

In conclusion, the results of this study supply evidence in support of utilizing siRNAs as a molecularly therapeutic method for Dox resistance to the treatment of human myelogenous leukemia, and further studies are warranted to deep understand the mechanisms of drug resistance in human myelogenous leukemia.

## CONFLICT OF INTEREST

There is no conflict of interest to declare in this research.

## Data Availability

All data, models and code generated or used during the study appear in the submitted article.
